# Novel model of cortical–meningeal organoid co-culture system improves human cortical brain organoid cytoarchitecture

**DOI:** 10.1038/s41598-023-35077-9

**Published:** 2023-05-14

**Authors:** Elmira Jalilian, Su Ryon Shin

**Affiliations:** 1 Department of Neurology, University of Michigan Medical Center, Ann Arbor, MI 48109 USA; 2 Division of Engineering in Medicine, Department of Medicine, Harvard Medical School, Brigham and Women’s Hospital, Cambridge, MA 02139 USA; 3 Department of Ophthalmology and Visual Sciences, University of Illinois at Chicago, Chicago, IL 60612 USA; 4 Richard and Loan Hill Department of Bioengineering, University of Illinois at Chicago, Chicago, IL 60607 USA

**Keywords:** Developmental biology, Neuroscience

## Abstract

Human cortical organoids (hCOs), derived from human induced pluripotent stem cells (iPSCs), provide a platform to interrogate mechanisms of human brain development and diseases in complex three- dimensional tissues. However, current hCO development methods lack important non-neural tissues, such as the surrounding meningeal layer, that have been shown to be essential for normal corticogenesis and brain development. Here, we first generated hCOs from a single rosette to create more homogenous organoids with consistent size around 250 µm by day 5. We then took advantage of a 3D co-culture system to encapsulate brain organoids with a thin layer of meningeal cells from the very early stages of cortical development. Immunostaining analysis was performed to display different cortical layer markers during different stages of development. Real-time monitoring of organoid development using IncuCyte displayed enhanced morphology and increased growth rate over time. We found that meningeal-encapsulated organoids illustrated better laminar organization by exhibiting higher expression of REELIN by Cajal–Retzius neurons. Presence of meningeal cells resulted in a greater expansion of TBR2 intermediate progenitor cells (IPCs), the deep cortical layer (CTIP2) and upper cortical layer (BRN2). Finally, meningeal-encapsulated organoids enhanced outer radial glial and astrocyte formation illustrated by stronger expression of HOPX and GFAP markers, respectively. This study presents a novel 3D co-culture platform to more closely mimic the in vivo cortical brain structure and enable us to better investigating mechanisms underlying the neurodevelopmental disorders during embryonic development.

## Introduction

The human brain is composed of multiple cell types, including neurons, astrocytes, microglia and oligodendrocytes. Current cerebral organoid systems derived from pluripotent stem cells, provide important in vitro models to recapitulate the developing human cortex, enabling us to study brain development in both healthy and diseased conditions^1–5^. However, there are several limitations to current in vitro model systems that can affect their reproducibility and applicability. One such limitation is the batch-to-batch variability that can arise due to differences in starting cell populations, culture conditions, and self-organizing properties of cells^6,7^. Addressing this limitation is crucial for developing organoids as reliable models for studying the human brain. Introducing single rosette-based approach, has allowed us to generate more consistent and reproducible organoids resulting in more homogenous and defined organoids that better mimic aspects of early brain development.

Another limitation of the organoid model is the lack of complete cellular diversity seen in the human brain^8^. While organoids can generate some of the cell types found in the human brain, not all cell types are represented. This may limit the proper signal transduction necessary to fully recapitulate the developmental and architectural aspects of human brain with appropriate complexity. To overcome this limitation, our approach is to incorporate meningeal cells as an important cell type that play pivotal role in brain development. Meninges a type of non-neuronal brain cell, are present from the very early embryonic stages of cortical development and appear to be necessary for normal corticogenesis and brain structure formation^9–12^. Recent studies have identified a non-parenchymal niche of NSCs population within the meninges that express neural precursor markers which are able to generate new neurons in vitro and in vivo^13,14^. During brain development, meninges play a crucial role in hCO development by releasing various morphogenic factors necessary for corticogenesis. In vitro studies have shown that meningeal cells can secrete FGF-2^15^, IGF2^16,17^, CXCL12^18–21^ and retinoic acid^22–24^ which are essential for proper corticogenesis. In vivo studies have also demonstrated that meninges secrete a verity of signaling molecules, including Sonic hedgehog (Shh), which plays a key role in the development of the ventral forebrain, and the bone morphogenetic protein (BMP) family, which regulates dorsal-ventral patterning of the neural tube^25^. Other factors secreted by the meninges include members of the fibroblast growth factor (FGF) family, Wnt, and Notch which are involved in the regulation of cell proliferation, differentiation, and survival in various developmental processes, including neurogenesis^1,26–29^. It is hypothesized that laminin-enriched areas in the meninges are essential for regulating the release of these factors. Therefore, the presence of meninges appears to be crucial for brain organoid development^30^.

Co-culture method for combining different brain regions within a single organoid tissue, has been shown previously to model complex interactions between different regions and study neurodevelopmental defects^31^. For instance, by using fluorescent reporters, it has been demonstrated that CXCR4-dependent GABAergic interneuron migrate from ventral to dorsal forebrain, and this has been used to study neurological diseases and test potential therapies^32^. However, no one has studied the use of meningeal cells in combination with brain organoids to investigate the effect of these cells on brain organoid development. In this study, we took advantage of a co-culture system to generate cortical brain organoids in combination with meningeal cells and studied the role of meningeal cells in the progression of laminar organization of early brain development in a model system.

## Materials and methods

### Human pluripotent stem cell culture

Three Induced Pluripotent Stem Cells (iPSCs) cell lines were used in this study. CC1, andCHD2were obtained from skin biopsies from different individuals and PCDH19-WT (commercially available foreskin fibroblasts) were previously generated by lab members and^20,21^. All iPSC cell lines were conducted with prior approval by the Institutional Review Board of Michigan Medicine. iPSCs were grown on 1:200 dilution of Geltrex-coated (Fisher Scientific) 30mm dish and in TeSR™-E8™ (StemCell Technologies). Cells were routinely passaged once a week and were used before the 35th passage number for all experiments.

### Meningeal cells

Human meningeal cells are derived directly from human leptomeninges and are commercially available (ScienCell catalog #1400). Leptomeningeal cells were cryopreserved at passage one and delivered frozen. Each vial contains > 5 × 10^5^ cells in 1 ml volume. HMC were characterized by immunofluorescence; they are all positive for fibronectin and negative for GFAP and α-smooth muscle actin. 5 × 10^5^ cells/ml, were purchased (1 ml/vial) in passage zero, expanded in meningeal cell medium MenCM consisting of 500 ml of basal medium, 10 ml of fetal bovine serum and 5 ml of meningeal cell growth supplement and 5 ml of penicillin/streptomycin (ScienCell catalog #1404) medium and were used up to passage number three.

### Forebrain organoid generation in in vitro

To generate cortical organoids, undifferentiated iPSC were used at the confluency of 70–80%. To start with plate preparation, 30 µl of 100% concentrated Geltrex was dropped in the middle of each well of a 12 well-plate and left in the incubator for 20 min to solidify as a bubble. Then, Geltrex diluted in DMEM/F12 (1:100) was then used to coat the rest of the surface around the Geltrex bubble. iPSC cells were then dissociated Geltrex coated plates by Accutase (Invitrogen, Carlsbad, CA, USA), and 8 × 105 cells in TeSRTM-E8TM medium were added around the bubble and not directly into the bubble and left in the incubator for 24 h to settle. Then the cell medium was changed into Neural induction medium 3N vitamin A containing 1µM dorsomorphin and 10 µM SB431542 as described in^22^ with some modifications. (3N medium details shown in Supplementary Data Table 1). Medium was exchanged every day with 2 ml of fresh medium. After 24 h single rosettes started to arise near the Geltrex bubble with the size of the rosettes observed to be increasing every day. The single rosettes nearest the Geltrex solid bubble were observably larger than other rosettes. After 5 days, these larger single rosettes were manually picked up and transferred individually into single wells of a 96 well round bottom plate with neural induction medium of 3N + vitamin A. Media was refreshed every other day, for 3 days.

### Co-culture of forebrain organoid with meningeal cells

On the fourth day after cell passage, meningeal cells were detached using Accutase (Innovative Cell Technologies) and added to single rosettes in suspension (iPSC cell day 8?). To determine the optimum number of meningeal cells to co-incubate with iPSC cells, half of the wells received 8 × 10^3^, meningeal cells, and one other half left with no meningeal cells as a control. The medium was 50% meningeal cell medium and 50% 3N + vitamin A. Meningeal media containing 2% FBS which was added half and half with 3N brought up the concentration of FBS down to 1%. This medium containing FBS was added for only 3 days (between days 9–11) and the same condition was considered for control condition. On day 9, the co-culture of organoids and meningeal cells were then added to an IncuCyte Zoom system (Essen BioScience, Ann Arbor, MI, USA) for incubation and imaging. Imaging was obtained every three hours for the first three days (days 9–11) and then every eight hours until day 42. Starting at day 12, the medium was refreshed once/day with 3N + vitamin A + 10 ng/ml BDNF + 10 ng/ml NT3. At the end of day 42, organoids were transferred to 48-well round bottom plates, with the same medium and placed in the incubator without IncuCyte, due to IncuCyte image size limitations. Organoids were fixed on days 42, 56 and 70 respectively for further imaging analysis.

### Tissue processing and immunohistochemistry

Organoids were fixed in cold 4% paraformaldehyde (PFA) in Dulbecco’s phosphate-buffered saline (DPBS) for 20 min and then washed twice in PBS for 5 min. Fixed organoids were then incubated overnight at 4 °C in 30% sucrose in PBS. The next day the organoids were removed from the sucrose and embedded and frozen in Tissue-Tek Optimal Cutting Temperature Compound (O.C.T., Sakura), cryo-sectioned at 20 µm thickness, and collected onto Superfrost Plus slides (Fisher Scientific). The slides were rinsed with 0.1% Triton-X100 (Sigma) for 20 min at room temperature to increase permeability and the sections were then incubated in ICC blocking buffer (PBS with 0.05% Tween-20, 5% normal goat serum and 1% BSA) for 1 h at room temperature. Next, sections were incubated with primary antibodies overnight at 4 °C. Antibody species, dilution, supplier name and catalog numbers are included in Supplementary Data Table 2. Cells were then washed with PBST (0.05% Tween 20 in PBS) three times for 10 min each followed by incubated with secondary antibodies diluted in the blocking solution for 90 min at room temperature. Slides were then washed one time with PBST for 10 min and incubated for 5 min in PBS with bisBenzimide to label nuclei. Finally, slides were washed three times for 10 min each in PBST and then mounted in Glycergel mounting medium, imaged using confocal microscopy (described below) and left overnight to dry for further imaging.

### Time-lapse imaging of organoid growth

The IncuCyte Zoom system is a platform that allows real-time imaging of spheroids using either label-free or dual-color fluorescence to observe morphology and study spheroid growth over time. Here, the system monitored rosette growth. The morphology and the average cross-sectional of each organoid at different time points over differentiation were observed and analyzed using the IncuCyte Zoom system software. All IncuCyte data were obtained from five independent experiments with at least six samples in each condition.

### Laser confocal microscopy–microscopy imaging

Sections were examined with a Leica TCS SP5 confocal laser-scanning microscope upright + inverted system (Leica Microsystems, Mannheim, Germany) using a sequential scanning procedure. Confocal images were taken with 10× and 20× lenses and data were analyzed by ImageJ. Confocal images were acquired using Zeiss LSM 700, LSM 780, or Zeiss LSM 800 confocal microscopes equipped with a motorized stage and Zen black or blue edition software. Tiled images were assembled using the Zen Tiles and Positions module. For brightfield imaging, an EVOS microscope (Advanced Microscopy Group) was used. All images were compiled in Adobe Photoshop, with image adjustments applied to the entire image and restricted to brightness, contrast, and levels. Images shown in figures as comparisons were obtained and processed in parallel using identical settings.

### Statistical analysis

Prism version 8.4.2 (GraphPad) was used for all statistical analyses. For all the other studies, the significant difference between test groups was evaluated using a two-tailed, unpaired Student’s t test. A P value  <  0.05 was considered statistically different. These experiments were repeated four times with three different cell lines. (CC1 n = 2, PCDH19 n = 1 & CDH2 n = 1). Immunostainings were performed for all of them. Although there were slightly differences in organoid size with different cell lines as shown in Supplementary data S1, all cell lines illustrated the same trend in differentiation stages and different markers. In this study, data were gathered and used from all three cell lines. At least n = 4 organoids were selected from each experiment (n = 4 biological replicates in total and n = 12 number of organoids that are mentioned in detail in figure legends). Thus, each graph represents the average and standard deviation after analysis of 18 individual organoids.

### Statement

All experiments and methods were performed in accordance with relevant guidelines and regulations. Informed consent was obtained from all subjects, and all methods were carried out in accordance with the relevant guidelines and regulations of REC.

## Results

### Cortical organoid differentiation derived from single rosettes

**Figure 1:**
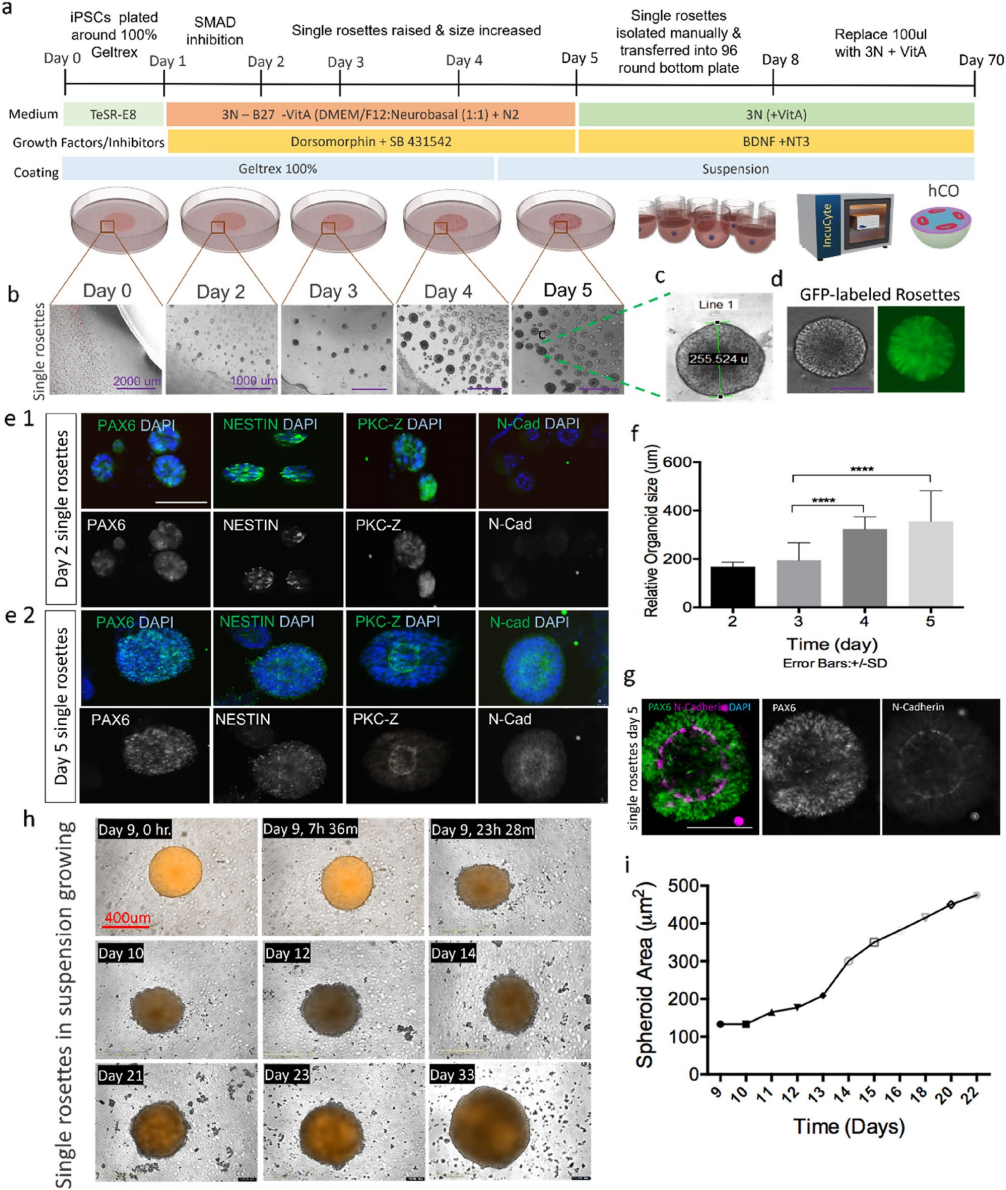
Generation and Characterisation of single neural rosettes*.* (**a**) Schematic diagram of culture method for generating human cortical organoids, (**b**) emergence and growth of single rosettes over five days at the interface between the bubble and dilute Geltrex, (**c**) representative image of a single rosette illustrates average size of each single rosette which is around 255 µm, (**d**) representative image of GFP-labelled rosettes, (**f**) quantitative analysis of single rosette growth in micrometres from day two to five. Data are represented as mean ± SD. Statistical analysis showed significant difference in size from days 3 to 4, and 4 to 5 (****p < 001, n = 4 independent experiments and n = 4 rosettes were selected from each experiment), (**e1,e2**) Immunostaining of single rosettes at day 2 and day 5 for NPCs (PAX6 and NETIN) and apical membrane (NCAD/APKC), (**g**) Single rosettes express PAX6 and N-cadherin, scale bar 100 µm, and (**h**) IncuCyte Zoom representative images of organoid growth in 96 well plates at different time points from day 9 to day 22. (**i**) Quantification of single rosettes size after the rosettes have acclimated to their 3D surrounding, starting at day 9 (n = 3 independent experiments from CC1 cell lines and n = 9 organoids in total). Error bars ± SD. Scale bar 400 µm.

We designed a method to generate single neural rosette structures from all three iPSCs (CC1, PCDH19-WT, CHD2) to replicate the cortical brain region. Neural rosettes by themselves lack the capability to spatially organize into distinct neuronal layers, but they are the starting point for 3D brain organoid^23^ and can be useful proxies for identifying potentially supportive cells for co-culture systems. For different growth factors and inhibitors, we modified the methods described by Yichen Shi^33^. Briefly, iPSCs were treated with dual SMAD inhibition (Dorsomorphin and SB431542), which is a well-established method to derive neural progenitor cells from iPSCs. A schematic diagram of the culture method is shown in (Fig. 1a). Single rosettes started to raise from day two near the interface between the solid Geltrex bubble and dilute Geltrex/DMEM (Fig. 1b) and grew over time. Data were taken from four independent experiments from GFP-labeled CC1 cells (Fig. 1d) and total number of n = 18 rosettes were analyzed. Mean rosette diameter for CC1 cell line on day 2 was 170 ± 20 µm and increased to > 250 µm by day 5 (Fig. 1c). Morphological observation showed well-defined lumen structures in the middle of the rosettes (Fig. 1c, g). Overall, single rosettes had > 1.8-fold increase in size from day 3 to 5 which was shown to be significant (p < 0005). (Fig. 1f). These data were confirmed with two additional iPSC lines (PCDH19 and CHD2). Data from these two lines are shown in (Supplementary Fig. S1). To characterize neural rosette emergence, we conducted a time course analysis on different neural stem/progenitor cell (NPC) markers. Only 2 days after dual SMAD induction, iPSC-derived rosettes started to show expression of neuroectodermal markers PAX6 and NESTIN NPCs and apical membrane marker PKC-Z (Fig. 1e1). At day five, well-defined polarized neuroepithelial-like structure was observed with distinguished expression of PAX6 and NESTIN NPCs in the ventricular zone (VZ) and strong expression of adherent junction markers of N-Cadherin and PKC-Z in the apical region which resembled neural tubes (Fig. 1e2, g).

Single rosettes were transferred to 96 well round bottom plates at day 5 and morphological changes were analyzed over 14 days using the IncuCyte Zoom system. Images were taken of each rosette every 12 h. Rosettes demonstrated size growth over time with rosettes nearly quadrupling in cross-sectional area from days 9–24 (Fig. 1h). Quantification of single rosettes size after the rosettes have acclimated to their 3D surrounding, starting at day 9 showed an increased in size diameter around > 800µm by day 24 (Fig. 1i). Our approach to generate cortical organoids from single rosettes appears to rapidly produce homogeneous organoids and yield well-defined organized neuroepithelial rosettes.

### Multiple progenitor zones mimic many futures of cortical development in vivo

**Figure 2:**
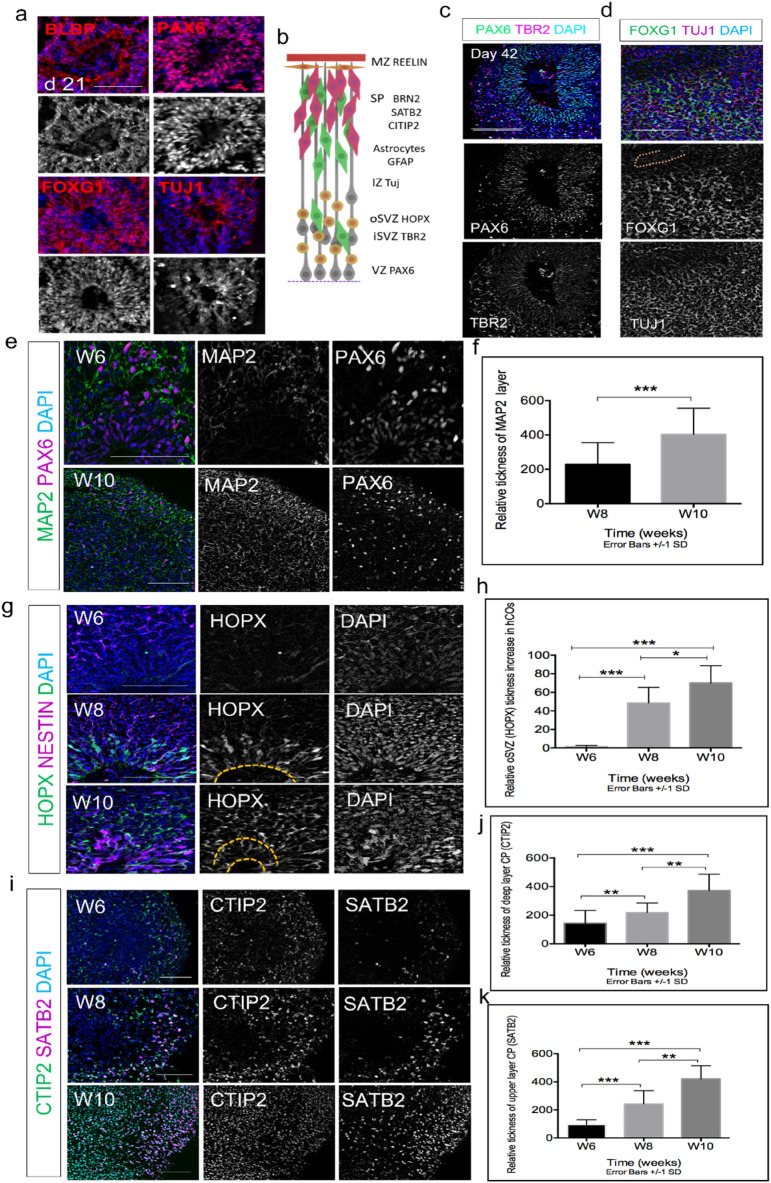
Immunostaining for region-specific markers and neuronal cell identities***.*** (**a**) Single rosettes immunoassayed at day 21 showed expression of general radial glia (RG) markers (PAX6 and BLBP), and forebrain marker FOXG1 and neuronal marker TUJ1. (**b**) Schematic expression of different brain regions. (**c**) Immunostaining of organoids at day 42 showed clear expression of RG (PAX6) in VZ, and IPC (TBR2) iSVZ. (**d**) Forebrain marker FOXG1 and neuronal marker TUJ1 identified in the VZ at day 42. (e) Cerebral organoids were co-stained for neuronal marker MAP2 and PAX 6 at day 42 (W6) and 70 (W10). (**f**) Quantification of MAP2 expression showed significant difference in W10 vs. W8 (P < 0.005), (**g**) Cerebral organoids were co-stained for neuroepithelial marker (NESTIN) and outer RG marker (HOPX). (**h**) Quantification of relative thickness of HOPX showed significant difference between three different time points, W6, W8 &W10 (P < 0.005), (**i**) cerebral organoids were co-stained for lower-layer neuron marker (CTIP2) and outer-layer neuron marker (SATB2) at days 42, 56 & 70, (**j,k**) statistical quantification of relative thickness of each layer showed significant between three different time points, W6, W8 &W10 (P < 0.005). (n = 4 biological replicates in total, n = 2 CC1, n = 1 PCDH19 and n = 1 CHD2, n = 12 total number of organoids were analysed). Error bars ± SD. Scale bar is 100 µm for all figures.

To further assess the protocol, we examined whether our method could generate different cortical regions and recapitulate human fetal cortex (Fig. 2b). We performed immunohistochemical analysis of specific cortical markers at different time points. At day 21, we observed clear expression of neuro-progenitor markers PAX6 and BLBP, forebrain marker FOXG1 and emergence of the neuronal marker TUJ1 (Fig. 2a). Previous studies have shown TBR2 as a critical factor for the specification of intermediate basal progenitor cells (IPCs) in a developing cortex. TBR2 is known to regulate morphogenesis of each cortical layer^34–36^. At day 42, cells within the organoid segregated into distinct PAX6 progenitors in VZ with prominent TBR2 IPCs in the subventricular zone (SVZ) (Fig. 2c). Also observed at day 42 were clear expressions of forebrain marker FOXG1 and neuronal marker TUJ1 (Fig. 2d). Furthermore, MAP2 expression, an indicator of neuron population, started at day 42 and significantly increased (P < 0.005) by day 70 (Fig. 2e, f).

We further assessed the expression of HOPX in our cell-culture system. In humans, SVZ, a key contributor to human neocortical growth, is divided into inner and outer sections called iSVZ and oSVZ, respectively^37^. Recent studies have shown the presence of outer radial glial cell (oRGC) is responsible for generation of most cortical neurons in the oSVZ layer similar to the developing human cortex at gestational weeks 15–20^38^. HOPX is one of the important characteristic markers of oRGCs^4,39^. We detected very little HOPX expression at day 42, however, clear emergence of HOPX at day 56 was detected in both the ventricular zone (VZ) and the SVZ. By day 70, the HOPX expression was significantly expanded (P < 0.005) and detected more in the outer subventricular zone (oSVZ-like region) which appeared to be separated from inner SVZ (iSVZ) region (Fig. 2g, h). Furthermore, at day 42, there is a clear expression of lower layer marker CTIP2 neurons formed above the VZ and SVZ resembling the preplate (PP), which expression significantly increased (*p* < 0.005) from day 56 (W8) to day 70 (W10) (Fig. 2g). Finally, the upper layer marker SATB2 emerged around day 42 and significantly, increased between W8 and W10 *(p* < 0*.*005) (Fig. 2g). Additional zoomed out images of entire human cortical organoids (hCOs) for all markers are shown in Supplementary Figs. S2 and S3. Together, these results demonstrate that our organoid system generated from single rosettes recapitulates human cortical development and patterned expression of crucial human developmental markers in vivo*.*

### Presence of meningeal cells significantly enhanced the morphology and phenotype of cortical organoids

**Figure 3:**
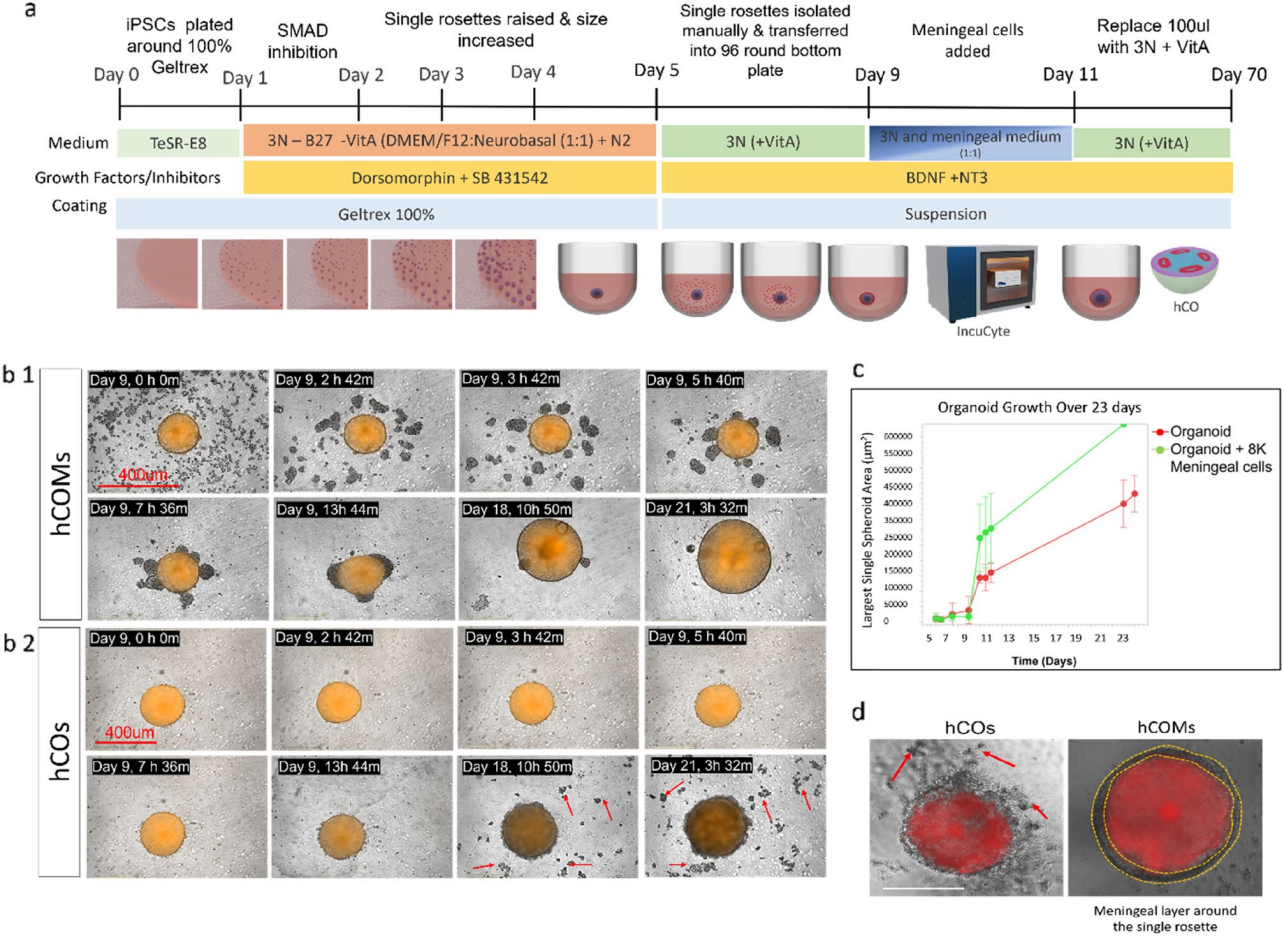
3D co-culture system for generation of human brain organoid*.* (**a**) Schematic illustrates the main steps of human cortical organoid (hCO) generation from iPSCs and cocultured with and meningeal cells starting at day 9. (**b1,b2**) Representative images of growth of organoids in IncuCyte Zoom showing 6 images on the day of coculture (day 9), and further images on day 18 and day 21 as well as representative images from hCO without meningeal cells. (**c**) Organoid growth in size using IncuCyte Zoom quantification software illustrated remarkable difference in growth of organoid between day 5–23. (**d**) Representative image to show the morphology of hCOMs vs hCOs using bright field microscope, scale bar 400 µm (n = 4 biological replicates in total, n = 2 CC1, n = 1 PCDH19 and n = 1 CHD2, n = 12 number organoids were analysed). Red arrows identify debris that is washed away during media change. Scale bar is 400 µm for brightfield images.

To better mimic the in vitro formation of human cortical organoid, we further used human meningeal cells in co-culture with our single rosette formation method to explore the effect of non-neuronal cells in assisting development of the human brain. For this experiment, human leptomeninges meningeal cells were used. Meningeal cells have been shown to be the source of developmental cues that regulate cortical differentiation and maturation^14^. Signals from different meningeal layers have also been shown to be crucial for proper formation of different brain regions^40–42^. For example, neurogenic signals such as retinoic acid from the middle meningeal layer regulates cortical neuron generation^22^. We wanted to see whether co-culture of meningeal cells, and the presence of guidance cues from these cells, can provide a better scaffold for RG migration and enhance the maturation of the neural organoid into separate discrete layers. To this end, we added single cell meningeal cells (~ 8000) to isolated single rosettes in round bottom 96 well-plates at day 9 and monitored their attachment in 2, 3 and 6 h. intervals by IncuCyte Zoom. It should be mentioned that different meningeal cell numbers were initially examined to define the optimum cell number for these experiments, and it was demonstrated that addition of more cells (16,000, 32,000 & 100,000 meningeal cells) resulted in smaller sized rosettes, perhaps because of the less space for organoids to grow (Supplementary Fig. S4a, b). Thus 8000 meningeal cells were chosen as the optimum cell number to co-culture with organoids. A schematic diagram of experimental procedure is shown in (Fig. 3a). We observed that most of the meningeal cells attach to and cover the single rosettes in the first 24 h (Fig. 3b1) and (Supplementary Video 1). The control condition without meningeal cells is shown in Fig. 3b2. Visual observations over 12 days clearly demonstrated that hCOs in presence of meningeal cells, which we called “hCOMs”, showed a superior-defined and more rounded spheroidal shape compared to hCOs (Fig. 3d) and (Supplementary Fig. S5). Furthermore, comparing the two conditions over real time monitoring the single organoids illustrated that more debris were released from the edge of the organoids into the medium in hCOs vs. hCOMs (red arrows Fig. 3b2). The debris were washed off by changing media. Moreover, further quantification analysis using IncuCyte Zoom software illustrated that the size of the organoid over 21 days remarkably increased (> 1.5-fold-change) in hCOMs compared to control hCOs (Fig. 3c). Data were analyzed from four separate experiments and a total number of 18 organoids for the CC1 cell line and results replicated with the use of two additional cell lines (PCDH19 and CHD2) in Supplementary Fig. S6. Together, these results show that our novel co-culture system using human iPSCs and meningeal cells together can efficiently enhance the morphology and phenotype of cortical organoid formation and increase their growth in a specific time course.

### Organization and marker expression of different progenitor zones and cortical neurons subtypes in hCOs vs hCOMs

**Figure 4:**
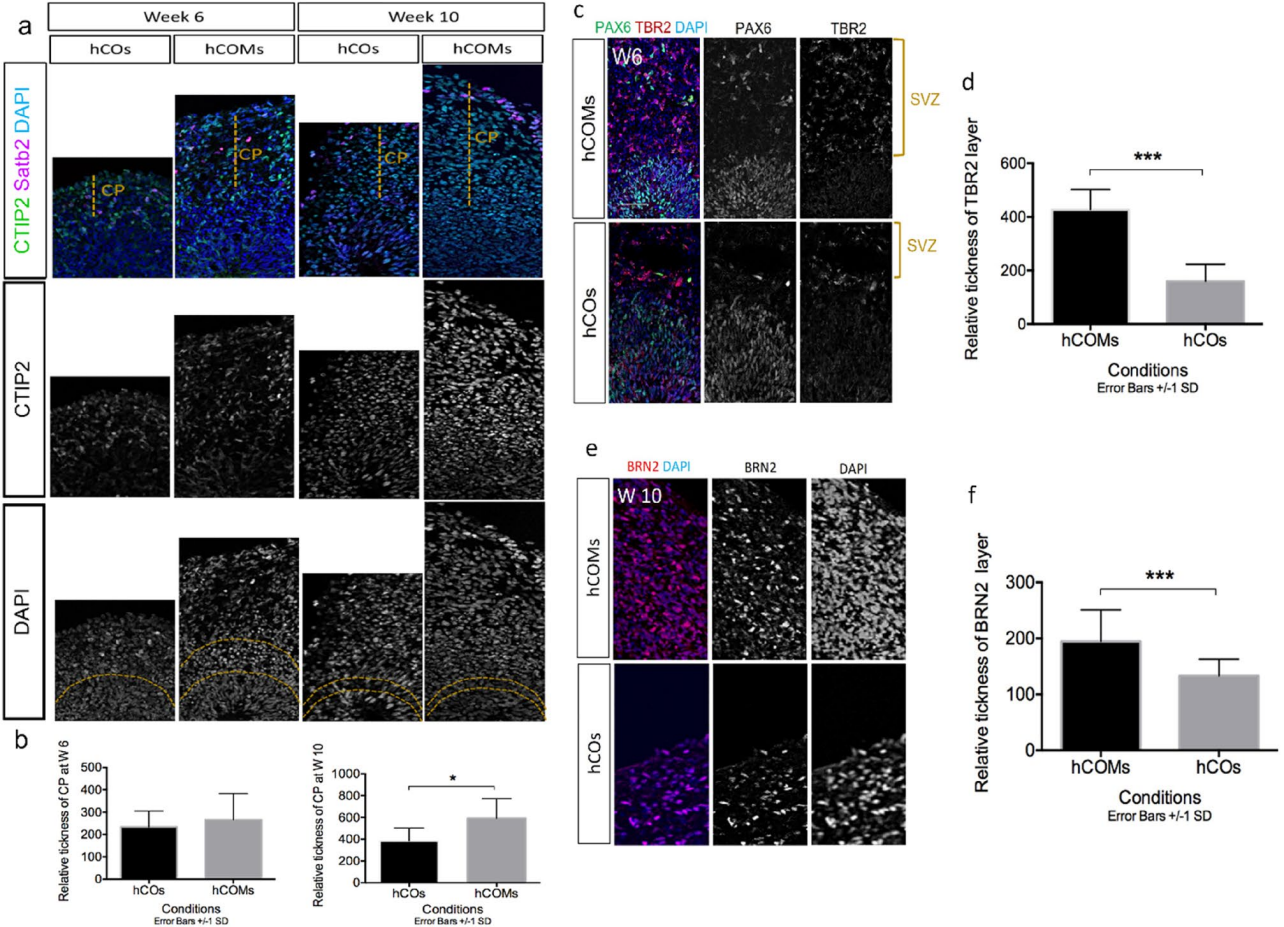
Co-culture system resulted in better development of corticogenesis, (**a**) immunostaining for lower-layer marker CTIP2 and upper-layer marker SATB2 in hCOMs versus hCOs after 6 weeks and 10 weeks. (**b**) Relative thickness of cortical plate (CP) at week 6 and week 10 in hCOs and hCOMs. For each cortical structure, three measurements were taken at 45° angles to obtain the mean value. Relative CP thickness is the ratio of CP thickness to total thickness from ventricular surface to pial surface. CP thickness was statistically significantly expanded after 10 weeks in hCOMs compared to hCOs, p < 0.05. Error bars ± SD, all scale bars: 100 µm, (**c**) cortical organoids immune-stained for PAX6 and IPC marker TBR2 showed comparatively expanded SVZ area in hCOMs vs. hCOs, and (**d**) statistical quantification of TBR2 expression showed significant difference in hCOMs vs. hCOs in W6 (P < 0.005). (**e**) Cortical organoids immune-stained for upper-layer marker BRN2 showed expanded and more defined area in hCOMs versus hCOs. (**f**) Statistical quantification of BRN2 expression showed significant difference in hCOMs vs. hCOs in W10 (n = 4 biological replicates in total, n = 2 CC1, n = 1 PCDH19 and n = 1 CHD2, n = 12 number of organoids were analyzed). Error bars ± SD. Scale bar, 100 µm.

To further compare cortical organoids in hCOs vs. hCOMs, we performed expression analysis of markers for different neuronal subtypes at different time points. By the end of weeks 6 and 10, we observed more neurons expressed deep-layer cortical neuron marker CTIP2 in a more expanded area in hCOMs compared to hCOs (Fig. 3a) and quantification measurements illustrated that this difference was significant after 10 weeks (Fig. 4b). At the end of week 6, we also observed well-defined VZ-like structures with packed PAX6 NPCs near the lumen and TBR2 IPCs formed above the VZ, reminiscent of the pre-plate (PP) in human cortical development in both hCOs and hCOMs. However, TBR2 IPCs were significantly more expanded in hCOMs (P < 0.005) (Fig. 4). BRN2 has been shown to have crucial roles in positioning of neocortical neurons^35^ and is a marker of layer III outer neurons. After observing the enhanced expansion of basal progenitor cells in the SVZ region, we further assessed the enlargement of neurons expressing BRN2 and we found that it was significantly expanded in the hCOMs compared with hCOs. (*p* < 0.005) (Fig. 4e,f). Zoomed out images of each figure are shown in Supplementary Fig S2 & S3. Together, our data reveal that cortical organoids grown in our novel 3D co-culture system illustrated well-defined cortical lamination.

### Presence of meningeal cells resulted in expansion of outer radial glial and marginal zone

**Figure 5:**
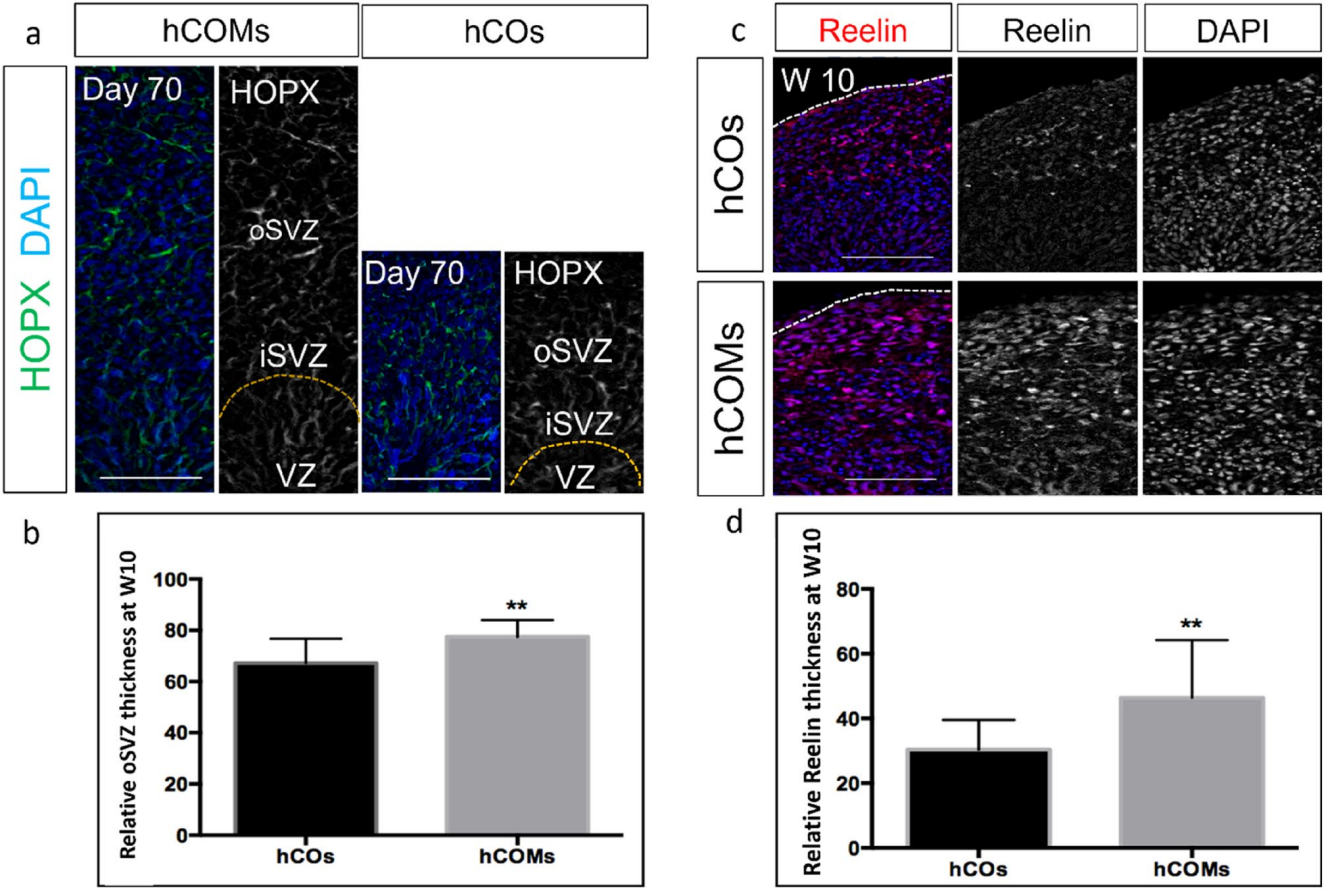
Organization and marker expression of different progenitor zones, (**a,b**) Sample images of immunostaining of oRGC markers HOPX after 10 weeks in hCOMs and hCOs and quantification of the relative thickness of the oSVZ area at week 10 illustrates significant increase in hCOMs compared to hCOs (p < 0005) (n > 10 cortical structures from 4 independent experiments). Error bars ± SD, all scale bars: 100 µm, (**c,d**) representative immunostaining images for preplate Cajal-Retzius marker REELIN after 10 weeks in hCOMs and hCOs and quantification of the relative thickness of the MZ area at week 10. For each cortical structure, three measurements were taken at 45° angles to obtain the mean. MZ thickness was statistically significantly expanded after 10 weeks in hCOMs compared to hCOs (p < 0.0005) (n = 4 biological replicates in total, n = 2 CC1, n = 1 PCDH19 and n = 1 CHD2, n = 10 number of organoids were analysed). Error bars ± SD, scale bars: 100 µm.

We performed further immunostaining assays to explore other neuronal markers for other layers. HOPX, a marker for the outer radial glial layer (oSVZ), was more expanded in hCOMs compared to hCOs (Fig. 5a). Image analysis showed this increase was significant (Fig. 5b). At day 70, we observed an enlarged area of REELIN expression at the marginal zone (MZ) in hCOMs versus hCOs. REELIN is present in the cortical MZ and is secreted by Cajal-Retzius neurons. REELIN is the key component in the evolution of the radial organization of the cortical plate^36^ and disruption of the expression of REELIN has shown to affect the proper laminar organization of the neocortex^37^. At day 70, we observed that thickness of layer expressing REELIN was remarkably enhanced in hCOMs compared with hCOs(Fig. 5c). To characterize this expansion, we measured the thickness of Reelin layer in both hCOs and hOMs and the data demonstrated a near twofold enhancement of marginal zone in the co-culture system (Fig. 5d). Zoomed out representative figures are shown in Supplementary Fig S3.

Next, we investigated whether signaling cues from meningeal cells can affect the enhancement of human NP proliferation on cortical formation in a 3D cerebral organoid system. Existing human genetic evidence strongly suggests that growth factor signaling such as FGF, regulates human cortical formation. In current study, both control and co-culture human organoids contained neuroepithelia organized in a stereotypic manner reminiscent of the early developing cortex, where Ki67+ NPs proliferated at the apical surface of the ventricular zone. Compared with controls, hCOMs harbored more Ki67+ cells at week 10 compared to week 8 where in control condition they decreased over time from week 8 to 10 (supplementary Fig S7). The enhanced proliferation was prolonged and most prominent at week 8 to 10 in hCOMs, however, in control hCOs, it declined over time. The developing cortex of humans and other gyrencephalic mammals harbor a larger VZ and SVZ, which are composed of substantially more radial glial cells and intermediate progenitors^43^, Therefore, the increased proliferation of IPC in our co-culture system suggests a better in vitro model of human corticogenesis with enhanced expansion capability that better recapitulates the human cortical brain, however, based on quantification data the difference in Ki67+ expression between hCOs and hCOMs was not significant.

We next asked how our co-culture system affects astrocyte differentiation. Astrocytes comprise the most numerous cell type in the mammalian brain and have been shown to have important roles in the CNS such as neurotransmitter recycling^44^, control of synapse formation^45^, and function^46^. Studies have also shown that meninges are the important sources of astrocyte-inducing factors in the developing brain. Our immunostaining results illustrated the expression of astrocytes at day 56 and day 70 in both hCOs and hCOMs which was to some degree more in hCOMs. (Supplementary Fig. S8).

## Discussion

The ability of hiPSCs to generate 3D brain-like constructs allows us to develop a novel personalized neurological modelling platform and investigate the mechanisms underlying human brain development and disease. Despite the current advanced technologies in brain organoid development, there are many technical challenges remaining. In this study, we established an efficient method based on single rosette formation from iPSCs which can reach to around 250 µm after 5 days. In just five days these rosette structures showed tube-like cytoarchitecture in the apical region expressing PKC-Z and N-CADHERIN and neural progenitor markers PAX6 and NESTIN in the basal region. Our results showed that these organoids express forebrain and neural markers after 21 days. Furthermore, our immunostaining analysis in different time courses could efficiently recapitulate the histological organization and pattern of expression for key developmental markers within the developing human cortex.

It is important to note that most of the experimental methods in the literature commence organoid fabrication by generating multiple rosettes from the beginning where each acts as an independent morphogenesis center^4,5,47^. Creating single rosettes has been recently attracted greater attention^48,49^. Single rosette-based protocols involve manually isolating and culturing well-formed neural rosettes, which are induced using growth factors and other signaling molecules. This approach allows for more consistent and reproducible organoid formation, resulting in more homogenous and defined organoids that better mimic aspects of early brain development. However, this approach is more labor-intensive and less scalable. In contrast, other techniques such as embryoid body-based protocols involve allowing pluripotent stem cells to aggregate into three-dimensional structures without manual manipulation. This approach is simpler and less labor-intensive, allowing for the generation of large numbers of organoids for high-throughput studies, but resulting in more variable organoids.^50,51^. Here we show that our organoid formation method can form single rosettes with less size variability over repeated experiments and increase the consistency in morphology and phenotype of forebrain organoids 250 ± 20 µm. However, it is important to note that although our method is based on single rosette formation in the first stage of differentiation, dissection of organoids at day 42 illustrated multiple VZs which show that as the hCOs mature, more VZs can be observed (supplementary Fig. 9).

We further developed our method with a 3D co-culture system using meningeal cells and assembled iPSC-derived hCOMs to assess the effect of co-culture with meningeal cells on morphology and phenotype of cortical brain organoids. Numerous studies have shown the crucial role of meninges as a source of several trophic factors including FGF2^15^, insulin-like growth factor^17,52^, CXCR12^19^ and retinoic acid^22,23^. Considering that previous studies have clearly shown the crucial role of meninges in developing neurons, intermediate progenitor production, and elongation of the neuroepithelium^22^, we wanted to take advantage of meningeal cells in our organoid culture to determine whether we can enhance corticogenesis and brain structure formation. In fact, we observed that presence of meningeal cells, and perhaps signalling factors from these cells, has significantly improved the differentiation and maturation of single rosettes and enhanced the morphology and cortical phenotype of our forebrain organoids. In vivo studies have shown that meninges secrete SDF1 that guides the tangential migration of Cajal-Retzius cells and cortical interneurons along the cortical marginal zone, ensuring their correct distribution during corticogenesis^42,53^. In our in vitro co-culture system, we observed significantly larger and clearly defined marginal zone areas shown by increased REELIN expression, which suggests that secretion of factors from meningeal cells generated a better-defined marginal zone and thus enhanced corticogenesis. We also observed expanded areas of IPC TBR2, deep layer marker CITIP2 and upper layer marker BRN2. Furthermore, the oSVZ layer which is the main feature of a human cortical organoid and separates it from rodents was much expanded and well-defined in our co-culture system. Moreover, we observed earlier maturation of astrocytes in our system and this is consistent with previous studies which showed meninges are important sources of astrocyte-inducing factors in the developing brain^54^. It is important to mention that, during the generation of organoid between days 9–11, organoids were exposed to 1% FBS. Studies has shown that, FBS can reactivate and increase the GFAP expression^55^. However, considering the fact that the first astrocytes will differentiate around day 40, which is approximately 15 full media changes after exposing to 1% FBS, this could suggest that mature astrocytes observed at day 70 were less likely generated as a consequence of medium effect. However, further experiments needed to find out whether increased expression of GFAP was observed due to presence of the FBS in the media or has expressed as a result of more differentiation and maturation. Also, it would be more informative for future studies, to use other astrocyte markers such as glutamine synthetase, BLBP (brain lipid-binding protein), S100β, and vimentin and CD49f. Furthermore, ideally, it is recommended to derive meningeal cells from the same pluripotent stem cell (PSC) lines used for generating cortical organoids to maintain the genetic and epigenetic compatibility necessary for proper differentiation and development. However, our research lab did not focus on differentiating meningeal cells from iPSCs, and it was not considered in our study. It is also important to consider the potential long-term effects of meningeal co-culture on hCO development which could include alterations in gene expression, cell proliferation, and differentiation. These effects may influence the development and maturation of hCOs and their capacity to model neurological disorders accurately. However, further research is needed to determine the specific long-term effects of meningeal co-culture on hCO development. Analyzing the functional properties of meningeal-encapsulated hCOs, including their ability to generate electrical activity and respond to external stimuli, could provide valuable information for understanding their potential as models for studying neurological disorders and drug discovery. Electrophysiological recordings and calcium imaging are potential methods for functional characterization of hCOs. These approaches will be considered in our future studies.

Our co-culture system has remarkably improved the laminar architecture of the rosette organoids and enabled better comparisons of organoids to native human tissue. Meningeal cells play crucial roles in brain development in vivo, including in the formation and maturation of the neocortex. In the case of hCOMs, the inclusion of meningeal cells in the 3D co-culture system may create a more physiologically relevant environment that better recapitulates the in vivo condition. Meningeal cells secrete growth factors and cytokines that regulate neural development, cell proliferation, and differentiation, which may promote the development of certain neuronal subtypes and their expression levels in hCOMs^50,56^. This may explain why hCOMs showed higher expression levels of certain neuronal subtype markers compared to hCOs. However, further studies are needed to fully understand the role of meningeal cells in the development of cortical organoids. Recent studies have shown that mTOR signaling can regulate the architecture of the developing human cortex by maintaining the cytoskeletal organization of oRG cells and the radial glia scaffold^53^. Thus, our co-culture system having a better defined and enhanced layer of oRGs offers a better in vitro model to recapitulate in vivo condition and improved cytoarchitecture and lamination of the developing brain.

Furthermore, future studies could consider adding an additional cell type, such as endothelial cells, in combination with meningeal cells to allow vascularisation of brain organoids. In tissue engineering, generating vascularized organoids is crucial for their sustained growth and to mimic the complex in vivo microenvironment. Pluripotent stem cell-derived organoids lack vasculature, and current strategies for organoid vascularization do not fully replicate in vivo co-development. To address this challenge, researchers developed a 3D printed microfluidic chip to co-culture human pluripotent stem cell-derived organoids with vascular cells in a spatially determined manner. On-chip pericytes and endothelial cells self-assembled into organized vascular networks and integrated with cerebral organoids, forming an integrated neurovascular organoid^57,58^. Furthermore, microfluidic channels made from degradable materials could further enhance organoid angiogenesis and better mimic the in vivo microenvironment^59^. This will provide a better platform for modeling a variety of neurological disorders, particularly neurodevelopmental disorders that involve abnormalities in cortical development, such as autism spectrum disorder (ASD), Attention Deficit Hyperactivity Disorder (ADHD), intellectual disability (ID), Developmental delay, schizophrenia, and epilepsy, and further future therapeutic applications. By recapitulating the complex cellular interactions that occur during cortical development, these models could provide new insights into the underlying mechanisms of these disorders, and could aid in the development of new therapeutic strategies.

## Supplementary Information

Supplementary Information.

## Data Availability

All relevant data are included in the manuscript. Materials, data, and protocols described within the paper are available upon reasonable request to the corresponding author.
