# Supplementary material for: Novel model of cortical–meningeal organoid co-culture system improves human cortical brain organoid cytoarchitecture
Source: Sci Rep. 2023 May 14;13:7809. doi: 10.1038/s41598-023-35077-9 (PMC10183460; doi:10.1038/s41598-023-35077-9)
Supplement: Supplementary file 1 — Supplementary Information. [file 41598_2023_35077_MOESM1_ESM.docx]

**Supplementary data**

**Novel model of Cortical-Meningeal Organoid Co-culture System Improves Human Brain Organoid Cytoarchitecture**

Elmira Jalilian^1.2,3^, Su Ryon Shin^4^

^1^Department of Ophthalmology and Visual Sciences, University of Illinois at Chicago, Chicago, IL 60612, USA

^2^Richard and Loan Hill Department of Bioengineering, University of Illinois at Chicago, Chicago, Illinois 60607, USA

^3^Department of Neurology, University of Michigan Medical Centre, Ann Arbor, Michigan 48109, USA

^4^Division of Engineering in Medicine, Department of Medicine, Harvard Medical School, Brigham and Women’s Hospital, Cambridge, MA 02139, USA

**
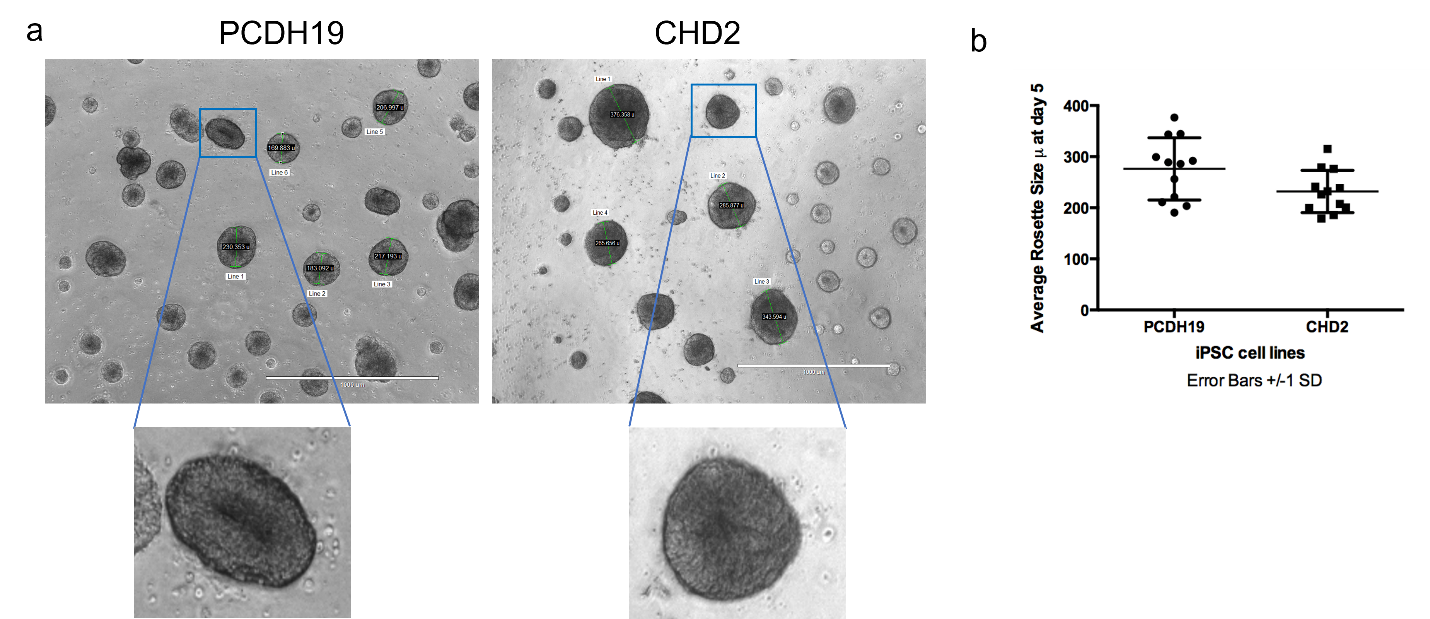
**

***Supplementary Figure S1***. **Brightfield images from single rosettes at day 5** (a) Images of rosettes from the iPSC cell lines PCDH19 and CHD2 illustrated increased in size of rosettes after 5 days (276.03 um in CC1 vs. 231.721 um in CHD2). (b) Quantification analysis of the average rosette size at day 5. (n=3 independent experiments and n=4 rosettes were selected from each experiment). Scale bar 1000 µm.


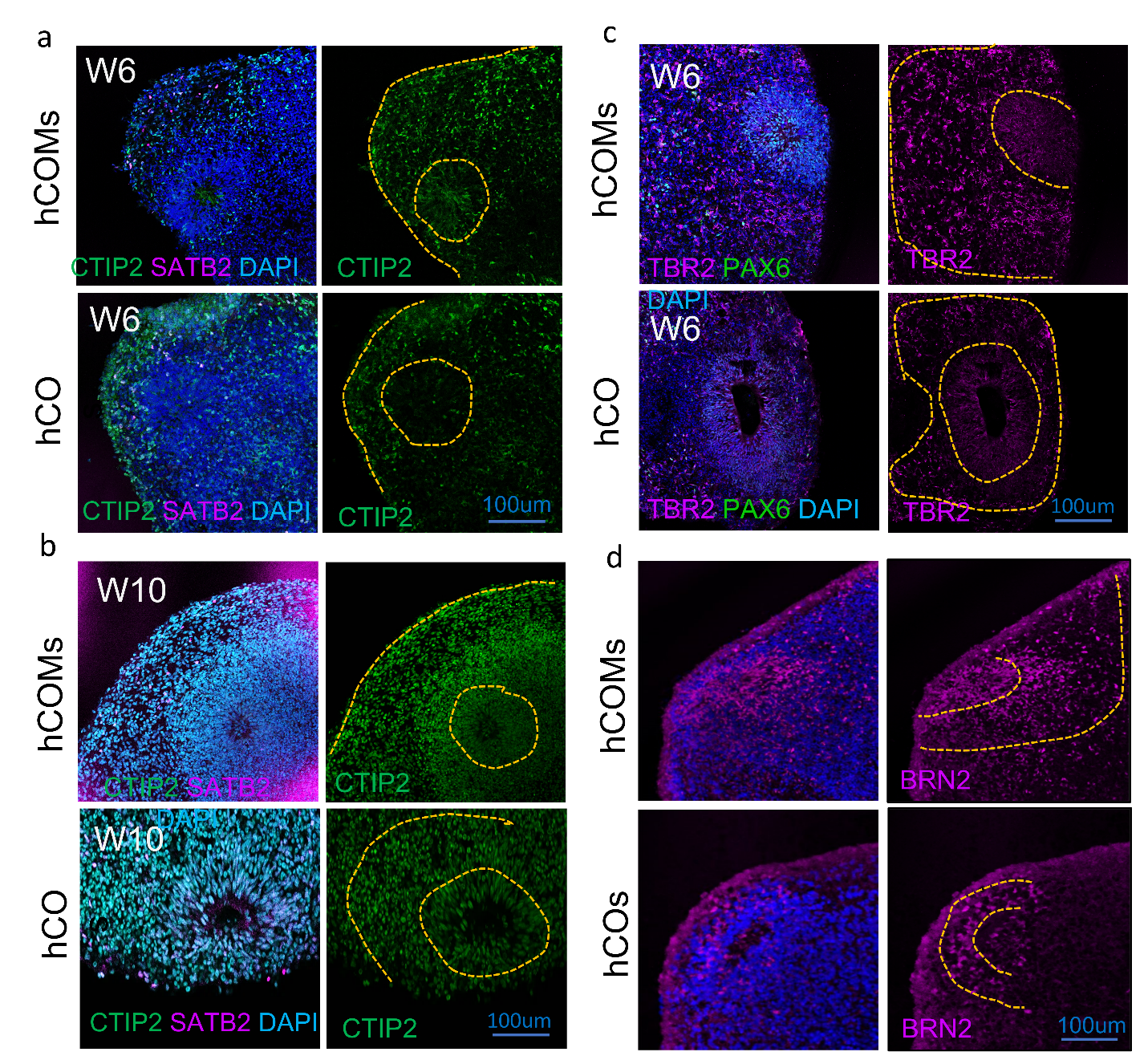


***Supplementary Figure S2.***  Zoomed out images of different expression markers in hCOs and hCOMs which show considerable expansion of CTIP2, TBR2 &BRN2 in hCOMs vs. hCOs. Yellow dotted lines are the area that illustrates the expression of specific marker. Scale bar 100 µm.


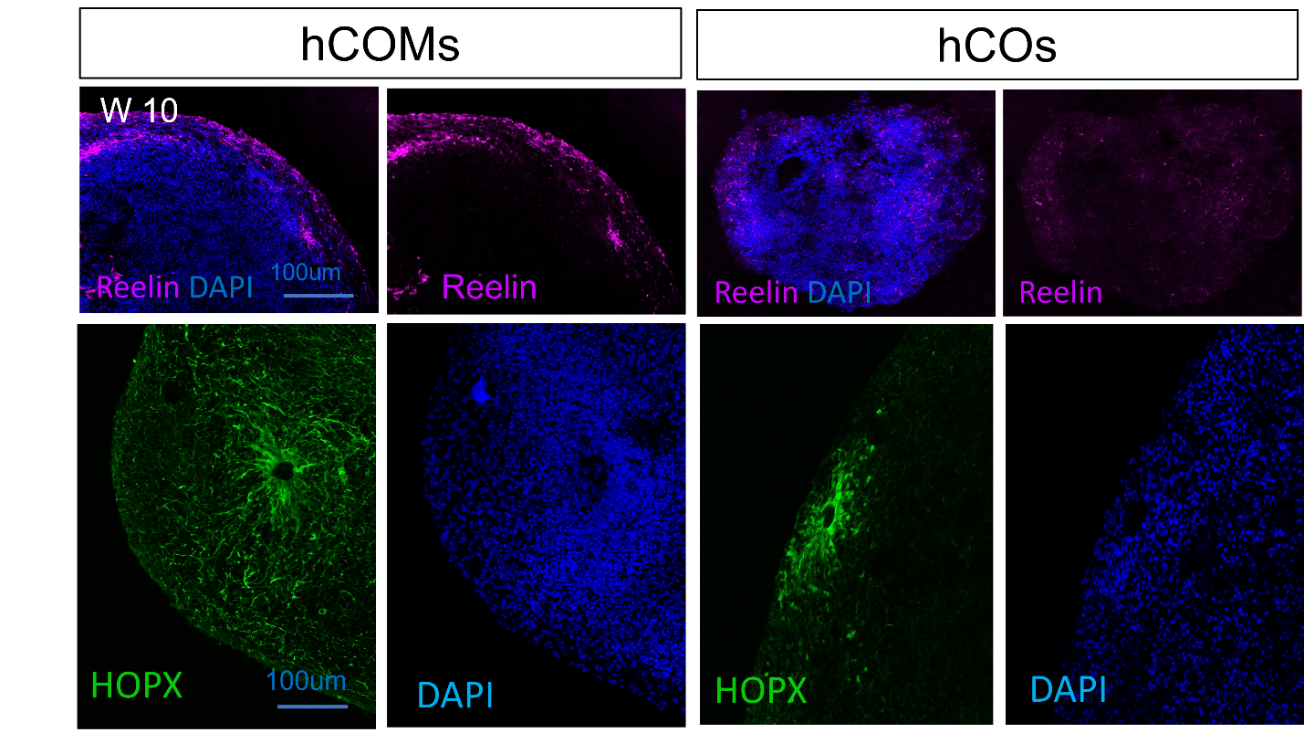


***Supplementary Figure S3***. Zoomed out images of different expression markers in hCOs and hCOMs which shows considerable expansion of Reelin and HOPX in hCOMs vs. hCOs. Scale bar 100 µm.


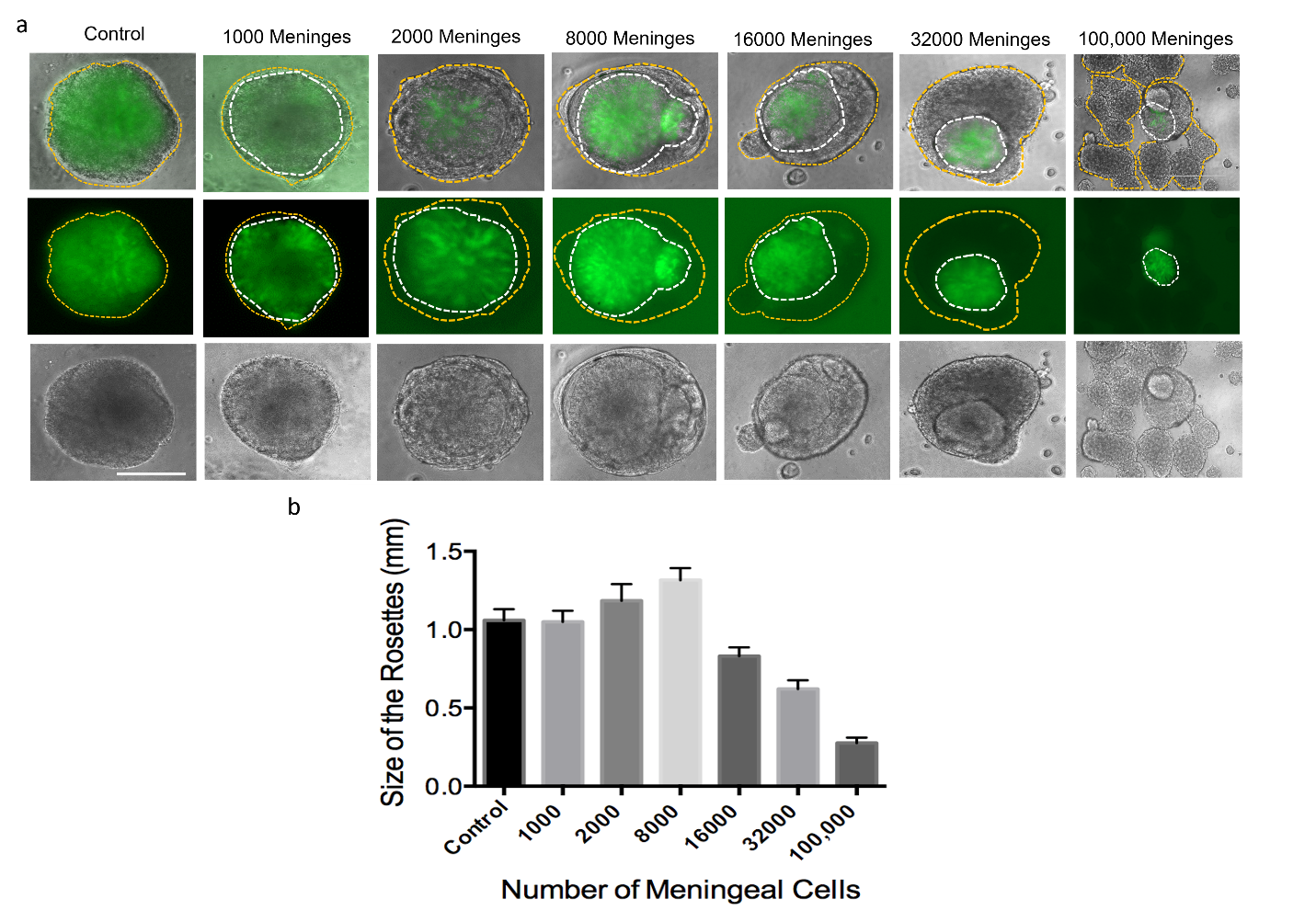


***Supplementary Figure S4:*** **Evaluation of optimum cell number for meningeal cells in co-culture system.** (a) Different meningeal cell numbers were added to single rosettes labelled with GFP (10002000, 8000, 16,000, 32,000 and 100,000) to define the optimum cell number for co-culture system. In results obtained after 5 days of adding meningeal cells at day 15 of differentiation, it was demonstrated that the highest cell numbers (16000, 32000 & 100,000 meningeal cells) resulted in smaller size of the rosettes. Thus 8000 was chosen as the optimum cell number. (b) Quantification analysis of rosette diameter demonstrated that higher number of meningeal cells (16,000, 32,000 and 100,000) resulted in smaller rosettes size diameter perhaps because of not enough space to grow and maximum rosettes size was observed in the condition with 8000 meningeal cells. Thus, rest of experiments were performed with 8000 of meningeal cells in the co-culture system. (n=2 biological replicate). Scale bar is 400 µm.


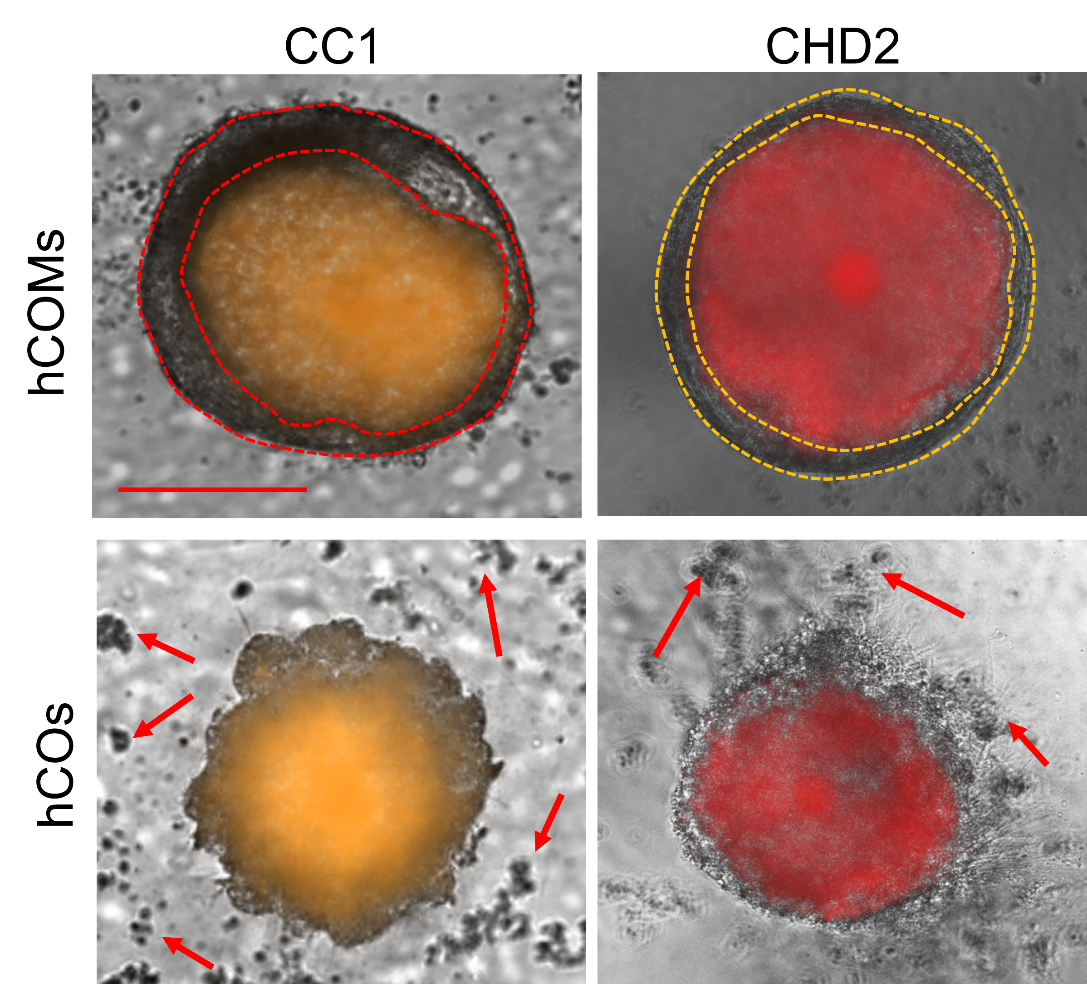


***Supplementary Figure S5***. **Meningeal layer around the single rosettes**. Representative images of hCOs and hCOMs from two cell lines CC1 & CHD2 (hCOs and hCOM) which shows enhanced morphology with rounder, more compact and clear edges of organoids in presence of meningeal cells. the bottom red arrows identify debris. Scale bar is 100 µm

**Supplementary Video 1:** Real-time monitoring of organoid development in presence of meningeal cells over 21 days displayed proper attachment of meningeal cells to organoids and illustrated enhanced morphology and increased growth rate over time. Images were collected every 2, 3 and 6 hrs and then every 8 hours for 21 days and analyzed using IncuCyte software. Scale bar, 400µm.


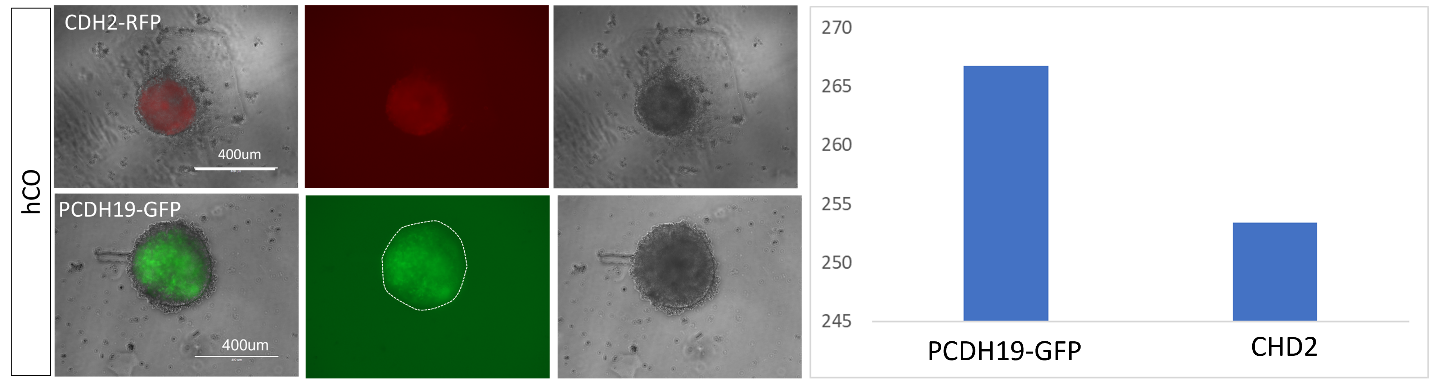


**Supplementary Figure S6**. Image of organoids at day 10 after transferring to 96-well plate from the iPSC cell lines PCDH19-GFP and CGH2-RFP. Scale bar 400 µm.


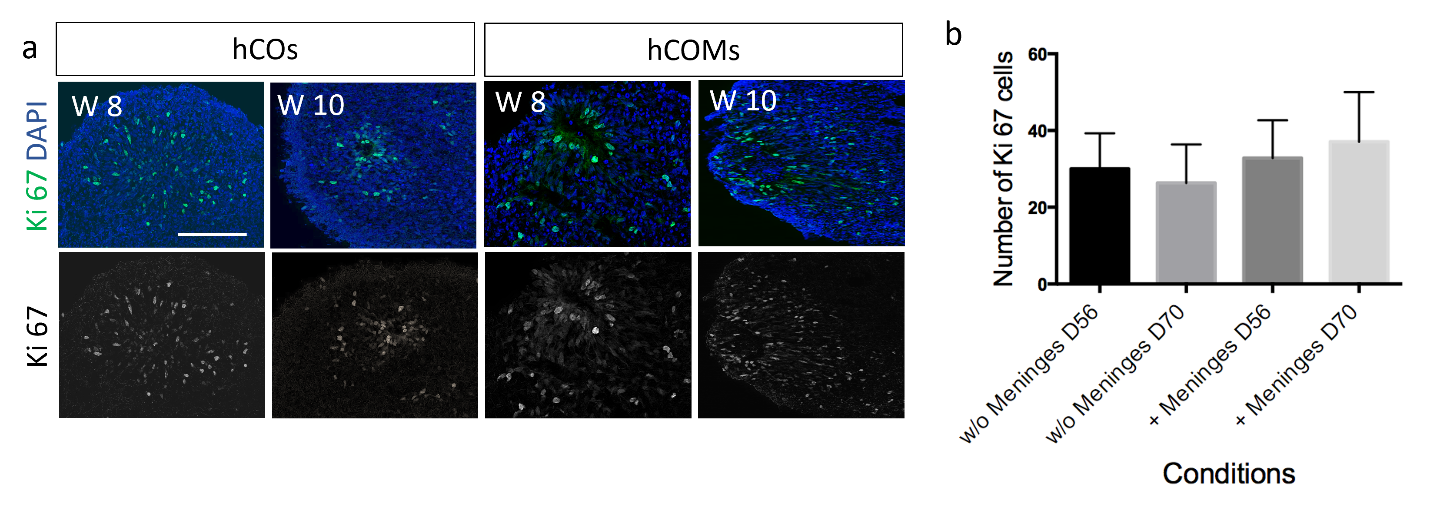


**Supplementary Figure S7.** (a) Representative images and quantification of Ki67 immuno-staining in hCOs and hCOMs. both control and co-culture human organoids contained neuroepithelia organized in a stereotypic manner reminiscent of the early developing cortex, where Ki67+ NPs proliferated at the apical surface of the ventricular zone. Compared with controls, hCOMs harboured more Ki67+ cells at week 10 compared to week 8 where in control condition they decreased over time from week 8 to 10. The enhanced proliferation was prolonged and most prominent at week 8 to 10 in hCOMs, however, in control hCOs, it declined over time (b) Quantification data illustrated that the difference in Ki67+ expression between hCOs and hCOMs was not significant. Scale bar, 100µm


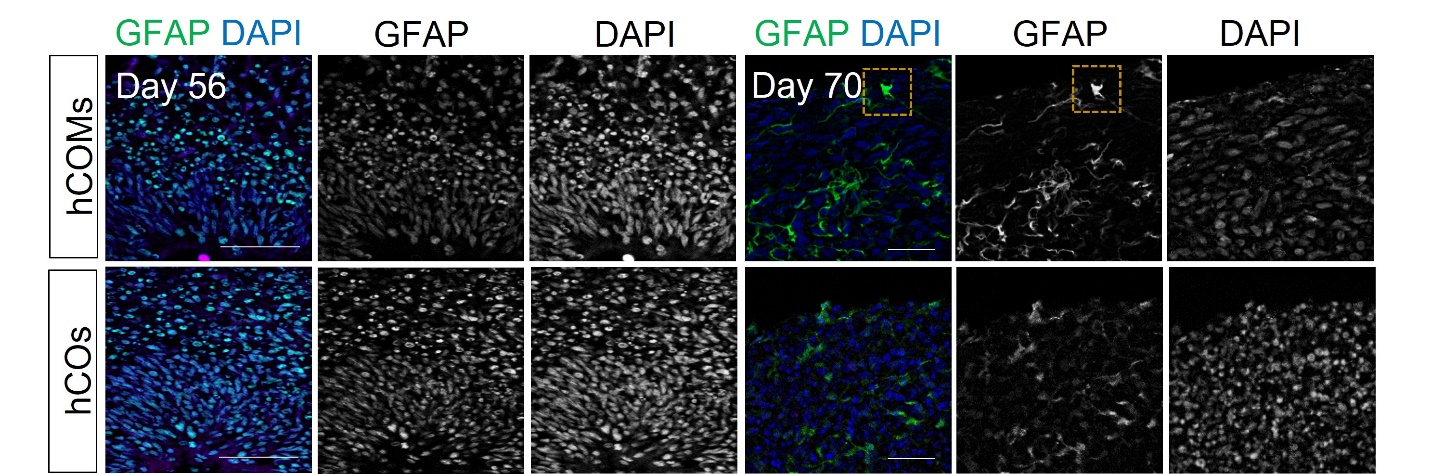


**Supplementary Figure S8:** Representative images of astrocytes marker GFAP after 8 and 10 weeks illustrated in hCOMs and hCOs.


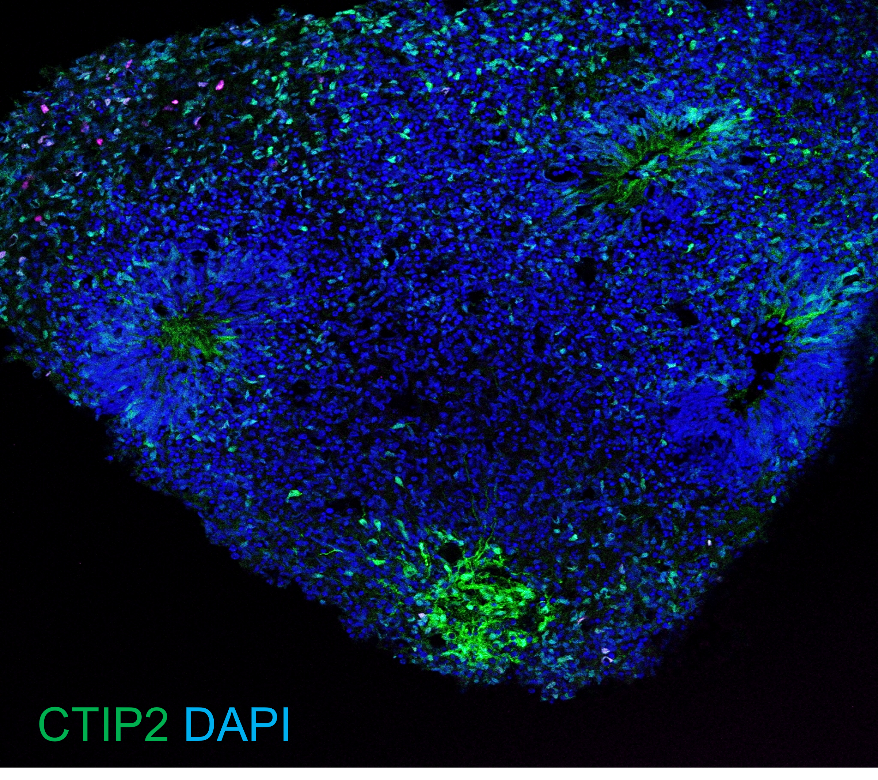


**Supplementary Figure S9:** Representative images shows multiple ventricular zones at day 42. As the astrocytes get mature, more VZ start to raise.

**Supplementary Data Table 1.**

*N2 medium*

| 500 mL | Component |
| --- | --- |
| 482.5 mL | DMEM/F12 with HEPES + L-Glut |
| 5 mL | NEAA |
| 5 mL | N2 |
| 2.5 mL | Glutamax |
| 2.5 mL | Pen/Strep |
| 250 uL | Insulin |
| 3.6 uL | BME |

*B27 medium*

| 500 mL | Component |
| --- | --- |
| 477.5 mL | Neurobasal medium |
| 5 mL | NEAA |
| 10 mL | B27 with or without Vitamin A |
| 2.5 mL | Pen/Strep |
| 2.5 mL | Glutamax |

**Supplementary Data Table 1.** Neural induction media (3N medium). The media is made 1:1 mixture of N2 and B27 media. To make 3N Mix 250 mL each N2 and B27 to make 3N, and sterile filter.

**Supplementary Data Table 2.**

| **Antigen** | **Species** | **DIlution** | **Company** | **Cat#** |
| --- | --- | --- | --- | --- |
| **BLBP** | Rb | 1(150) | Millipore | ABN14 |
| **BRN2** | Rb | 1(500) | Genetex | GTX114650 |
| **CR -50 (Reelin)** | M | 1(250) | Private | --- |
| **CTIP2** | Rt | 1(300) | Abcam | ab18465 |
| **FOXG1** | Rb | 1(1000) | Abcam | ab18259 |
| **GFAP** | M | 1(500) | Sigma | G3893 |
| **GFAP** | Rb | 1(2000) | Dako/Agilent | Z0334 |
| **HOPX** | Rb | 1(500) | Sigma | HPA030180 |
| **MAP2 (ab)** | M | 1(500) | Sigma | M2320 |
| **N-Cadherin** | M | 1(500) | Life technologies | 33-3900 |
| **Nestin** | Ms | 1(300) | Millipore | MAB5326 |
| **PAX6** | Rb | 1(1000) | MBL | PD022 |
| **SATB2** | M | 1(100) | Abcam | ab51502 |
| **Tuj1** | M | 1(1000) | Covance | MMS-435p |

**Supplementary Data Table 2.** List of antibodies we used in this study.
